# Risk prediction for 12-month post-prostatectomy urinary incontinence to support survivorship care: development and same-center temporal validation of a perioperative model

**DOI:** 10.3389/fpubh.2026.1968281

**Published:** 2026-10-21

**Authors:** Lang Tang, Ming Hao, Mengru Luo, Shizhu Li

**Affiliations:** 1 Department of Urology, The Second Affiliated Hospital of Guizhou University of Traditional Chinese Medicine, Guiyang, Guizhou, China; 2 Department of Urology, Guiyang First People’s Hospital, Guiyang, Guizhou, China

**Keywords:** LASSO, penalized logistic regression, prediction model, radical prostatectomy, rehabilitation, risk stratification, temporal validation, urinary incontinence

## Abstract

**Background:**

Persistent post-prostatectomy urinary incontinence (PPI) is a survivorship problem after radical prostatectomy with continuing implications for daily function, rehabilitation, follow-up, and supportive care. Risk stratification could help target these services, but uncommon events constrain model complexity and require evaluation beyond discrimination.

**Objective:**

To develop a perioperative model for PPI 12 months after catheter removal using LASSO-penalized logistic regression, compare it with a constrained gradient boosting machine (GBM), and examine temporal performance in a later same-center cohort.

**Methods:**

The development cohort comprised 726 patients with 51 PPI events (7.0%) and 19 candidate predictors. LASSO tuning used the one-standard-error rule within resampling, and the strategies were compared by nested repeated stratified five-fold cross-validation. The selected LASSO model was refitted to the full development cohort and applied without coefficient updating or recalibration to 465 later patients with 38 events (8.2%). Evaluation included ROC-AUC, average precision, Brier score, calibration-in-the-large (CITL), calibration slope, threshold-dependent measures, and decision-curve analysis.

**Results:**

Eight original predictors were retained: age, preoperative International Prostate Symptom Score, ln(1 + prostate-specific antigen), operative time, postoperative hospital stay, smoking history, hypertension, and neurovascular bundle preservation. Nested internal validation yielded a ROC-AUC of 0.913 (95% CI 0.871–0.946), average precision of 0.542 (0.404–0.675), and Brier score of 0.046 (0.035–0.058) for LASSO, versus 0.896, 0.488, and 0.048 for constrained GBM. In the temporal cohort, ROC-AUC was 0.836 (0.763–0.900), average precision 0.409 (0.277–0.564), and Brier score 0.062 (0.045–0.081). CITL was 0.445 (0.012–0.808) and the calibration slope 0.850 (0.621–1.174), indicating average underprediction. At the development-derived threshold of 0.128, sensitivity was 0.500 and specificity 0.913. Decision-curve analysis indicated potential net benefit across thresholds of 2–30%.

**Conclusion:**

The LASSO model retained useful temporal discrimination but underestimated absolute risk, and the development-derived cutoff did not transport well. It is therefore better suited to continuous risk estimation for survivorship follow-up and rehabilitation planning than to a fixed binary triage rule. Independent geographic validation, local calibration assessment, and prospective evaluation of clinical impact are required before routine use.

## 1 Introduction

Radical prostatectomy (RP), including robot-assisted radical prostatectomy (RARP), is an established treatment with curative intent for localized prostate cancer (1). As long-term cancer control improves, functional survivorship has become increasingly important to patients and care systems. Urinary incontinence after prostatectomy is particularly consequential because persistent leakage can restrict activity, affect psychological and social well-being, reduce health-related quality of life, and create continuing needs for continence products, rehabilitation, outpatient review, and other supportive services (2, 3). Recovery has been linked to patient age, preoperative lower urinary tract symptoms, disease and anatomic characteristics, and neurovascular bundle preservation (4, 5). From a survivorship and service-planning perspective, the practical question is whether routinely available perioperative information can provide a sufficiently reliable estimate of longer-term continence risk to support proportionate follow-up and rehabilitation planning.

Previous work has used conventional regression, nomograms, random forests, support-vector machines, gradient boosting, and deep-learning-derived surgical metrics to predict continence recovery after RP (6–8). More flexible algorithms can model nonlinear effects and interactions, but flexibility alone does not guarantee better clinical prediction (9). This issue is especially important when the number of events is small relative to the number of candidate parameters, because aggressive feature selection or complex learners may yield optimistic and unstable estimates (10, 11). A simple random split can also waste scarce event information, while ROC-AUC by itself says little about the numerical accuracy of predicted probabilities or the positive predictive value achieved in a low-prevalence setting (12). For a model intended to inform allocation of follow-up or rehabilitation resources, these limitations are directly relevant because poorly calibrated or unstable estimates can misclassify the intensity of support that patients may require.

A useful risk-stratification model must therefore do more than rank patients correctly. Calibration and the Brier score address whether estimated risks are credible on an absolute scale (12, 13), and precision-recall measures add information when the outcome is uncommon (14). Testing a locked model in patients treated later at the same institution can reveal temporal changes that internal resampling cannot capture (15). Such evaluation is still not equivalent to geographic external validation: differences in case mix, surgical practice, and care pathways can alter both calibration and the performance of any fixed decision threshold (15). These considerations are important when prediction is proposed as an aid to survivorship care, because service thresholds should reflect local event rates, care pathways, consequences of missed high-risk patients, and available rehabilitation capacity rather than discrimination alone.

Against this background, we developed a perioperative prediction strategy centered on LASSO-penalized logistic regression and evaluated its performance over time. All 19 candidate predictors were supplied to both LASSO and a deliberately constrained gradient boosting machine (GBM), thereby comparing a sparse linear approach with limited nonlinear flexibility. Candidate strategies were assessed by nested repeated cross-validation, after which the selected model was fitted to the entire development cohort and applied unchanged to a subsequent same-center cohort. The proposed role is postoperative risk stratification to inform counseling, functional follow-up, pelvic-floor rehabilitation planning, and prioritization of supportive-care resources. The model is not intended for diagnosis, causal inference, selection of surgical treatment, or stand-alone determination of access to services.

## 2 Materials and methods

### 2.1 Study design, setting, and participants

This retrospective prediction-model study used two non-overlapping cohorts from the same clinical setting. Patients undergoing RP/RARP between 1 January 2020 and 31 December 2023 formed the development cohort. Potentially eligible patients treated between 1 January 2024 and 30 June 2025 formed the same-center temporal validation cohort. Follow-up for the prespecified outcome at 12 months after catheter removal continued through July 2026. The analysis included only patients for whom the 12-month outcome had been ascertained.

Eligibility required pathologically confirmed prostate cancer, a first RP/RARP, completion of the required preoperative assessment, and availability of essential clinical information and 12-month follow-up. Patients were excluded for preoperative urinary incontinence, previous pelvic radiotherapy, severe neurologic or psychiatric disease that precluded outcome assessment, loss to follow-up before 12 months, or missing essential data. Six of 732 candidate development-period records were excluded because of data anomalies, leaving 726 patients in the development cohort. During the temporal-validation period, 476 potentially eligible patients were identified. Of these, 1 was excluded because of preoperative urinary incontinence, 2 because of previous pelvic radiotherapy, 1 because of severe neurologic or psychiatric disease precluding outcome assessment, 3 because of missing essential data, and 4 because 12-month outcome ascertainment was unavailable. The remaining 465 patients had complete candidate-predictor information and completed 12-month outcome ascertainment and comprised the temporal validation cohort.

During an earlier modeling stage, inspection of the temporal cohort was limited to baseline descriptive characteristics, outcome prevalence, basic data-quality indicators, and exploratory performance outputs from a preliminary model. None of these observations were used to estimate, tune, update, or recalibrate the final LASSO model. Predictor selection, data preprocessing, penalty tuning, coefficient estimation, threshold determination, and final model fitting were conducted exclusively using the development cohort. The resulting model was fully locked before application to the temporal validation cohort, and neither its coefficients nor its threshold was re-optimized using temporal-cohort data. Because prior awareness of temporal-cohort characteristics and preliminary performance results could nevertheless have indirectly influenced subsequent analytical decisions, we regard this evaluation as same-center temporal validation rather than as a fully untouched confirmatory prospective or geographic external validation.

Because this retrospective study included all eligible patients during the defined development period, no *a priori* sample-size calculation determined the available cohort size. The 19 original candidate predictors corresponded to 21 candidate predictor parameters before penalization because surgical approach and neurovascular bundle preservation were each represented by two indicator parameters. We therefore performed a *post hoc* sample-adequacy sensitivity assessment using the Riley et al. (11) framework for binary-outcome prediction models. The assessment used 21 candidate parameters, the observed development-cohort event proportion of 0.070, a target global shrinkage factor of 0.90, a maximum absolute difference of 0.05 between apparent and adjusted Nagelkerke R-squared, and an absolute margin of error of 0.05 for the overall outcome proportion. Because a directly comparable externally validated Cox-Snell R-squared estimate was unavailable, anticipated model fit was examined across an illustrative Nagelkerke R-squared sensitivity range of 0.10–0.30; a value of 0.15 was used as the primary scenario. This assessment was intended to quantify sample adequacy and was not treated as a retrospective power calculation.

### 2.2 Outcome definition and follow-up

A uniformly trained urology nursing team conducted follow-up with the International Consultation on Incontinence Questionnaire-Short Form (ICIQ-SF), a validated patient-reported measure of urinary incontinence symptoms and impact (16). Time zero for prediction was catheter removal, and the primary endpoint was PPI at 12 months. We defined PPI as ICIQ-SF ≥ 1 and complete symptom-free continence as ICIQ-SF = 0. Because definitions of continence after radical prostatectomy vary across studies (3), this threshold was used specifically to identify any residual self-reported incontinence symptom and should not be regarded as a universally validated diagnostic cutoff.

ICIQ-SF assessments at 3 and 6 months were excluded from both the predictor set and outcome definition for this analysis. The 12-month endpoint was defined identically in the development and temporal cohorts.

### 2.3 Candidate predictors and preprocessing

The source database provided 19 candidate predictors covering demographic characteristics, baseline urinary function, disease-related features, perioperative factors, and imaging or anatomic measurements: age, body mass index, smoking history, alcohol history, hypertension, diabetes, preoperative IPSS, preoperative PSA, prostate volume, history of medications affecting urination, surgical approach, operative time, estimated blood loss, neurovascular bundle preservation, pelvic lymph-node dissection, catheter duration, postoperative hospital stay, membranous urethral length, and apical urethral injury score. Neurovascular bundle preservation was categorized as bilateral, unilateral, or none, with bilateral preservation as the reference. Surgical approach was classified as robotic, laparoscopic, or open.

Preoperative PSA was transformed as ln(1 + PSA). In each resampling iteration, means and standard deviations estimated from the training data were used to standardize continuous variables, and those same constants were applied to the corresponding validation data. Binary variables remained coded 0/1, and multicategory variables were represented by reference coding. Preprocessing was therefore contained within the modeling workflow and did not use information from validation observations. No imputation was necessary because candidate predictors and the primary outcome were complete in both analysis cohorts.

The intended prediction time was catheter removal. Catheter duration and postoperative hospital stay were therefore eligible perioperative predictors because these quantities were available by the intended prediction point. The model is intended for risk estimation at or around catheter removal rather than for purely preoperative decision-making.

### 2.4 LASSO variable selection

LASSO-penalized logistic regression provided simultaneous coefficient shrinkage and variable selection (10). We evaluated 24 logarithmically spaced values of the inverse regularization parameter C, ranging from approximately 0.003 to 10. For the final model, C was tuned in the development cohort by repeated stratified five-fold cross-validation with 10 repeats and log loss as the optimization criterion. Under the one-standard-error rule, the selected value was the strongest penalty whose mean cross-validated log loss lay within one standard error of the minimum. An original predictor was deemed retained if at least one of its coefficients was non-zero in the final fit to the full development cohort.

Selection stability was explored descriptively by refitting the LASSO procedure in 1,000 patient-level bootstrap samples while holding the selected penalty fixed. For each original predictor, we recorded the proportion of bootstrap samples in which at least one associated coefficient was non-zero. These retention frequencies were used only to describe stability and did not determine the final predictor set.

### 2.5 Candidate models and nested internal validation

The same 19 starting predictors were used for two candidate strategies: LASSO-penalized logistic regression and a constrained gradient boosting machine (GBM) (17). To limit model flexibility in view of the small event count, GBM was prespecified with 100 trees, a learning rate of 0.05, maximum depth of 2, at least 20 observations per leaf, and a subsampling fraction of 0.8.

Internal performance was quantified by five repeats of stratified five-fold outer cross-validation. Within each outer training partition, LASSO tuning was repeated by inner stratified five-fold cross-validation using the one-standard-error rule. Consequently, preprocessing, penalty selection, and variable selection were completed without access to the corresponding outer validation fold. This nested structure limits the optimism that arises when tuning and performance estimation share the same resampling loop (18). The constrained GBM was fitted to the identical 19 predictors, with preprocessing estimated exclusively from each outer training set. Every participant served once as an outer validation observation in each repeat; the five out-of-fold probabilities obtained per participant and model were then averaged to form a single internal-validation prediction.

LASSO was the primary modeling strategy, and the constrained GBM served as a comparator representing limited nonlinear flexibility. Candidate strategies were compared descriptively using average precision, ROC-AUC, Brier score, calibration, model complexity, and clinical interpretability. No single numerical superiority margin was defined in advance. The final model choice therefore considered out-of-fold predictive performance together with parsimony and interpretability.

### 2.6 Final model fitting, locking, and temporal validation

LASSO-penalized logistic regression was retained after the internal comparison. Its final penalty was chosen by the repeated development-cohort procedure described above, and one model was then fitted to all 726 development participants. The intercept, coefficients, and preprocessing constants from this fit were locked before evaluation in the temporal cohort.

The locked model was applied to all 465 temporal-cohort patients as specified, with no retuning, coefficient updating, variable reselection, recalibration, imputation, or threshold re-optimization. No element of the revised LASSO model was altered on the basis of temporal-cohort data.

For clarity, LASSO was used in three distinct ways. First, within nested cross-validation, preprocessing, penalty tuning, and variable selection were repeated inside the training data so that out-of-fold predictions evaluated the complete modeling procedure. Second, after the candidate-model comparison, one final LASSO model was fitted to the full 726-patient development cohort to obtain the published locked equation. Third, bootstrap refitting was used only to describe predictor-retention stability. The bootstrap selection frequencies did not determine which predictors were included in the final model.

### 2.7 Performance, calibration, threshold analysis, and clinical net benefit

Discrimination was summarized by ROC-AUC. Because PPI was uncommon, precision-recall performance was also reported as average precision, for which event prevalence provides a no-skill reference (14). The Brier score summarized overall probability error. Calibration was examined using calibration-in-the-large (CITL; ideal value 0), calibration slope (ideal value 1), and a plot combining a logistic calibration relation, bootstrap pointwise uncertainty, and quintile-based observed-versus-predicted anchors (12, 13).

For internal validation, each participant contributed one outer out-of-fold predicted probability in each repeat. The five out-of-fold probabilities were averaged to obtain one internal-validation probability per participant. Calibration slope was estimated as beta in the logistic calibration model logit[P(Y = 1)] = alpha + beta x logit(p_hat), where p_hat was the averaged out-of-fold probability. Calibration-in-the-large (CITL) was estimated as the calibration intercept with logit(p_hat) entered as an offset (equivalent to fixing the calibration slope at 1). The same definitions were used for temporal validation using the locked-model probabilities.

For the final LASSO model, 95% confidence intervals for internal out-of-fold and temporal performance were obtained from 1,000 patient-level bootstrap resamples while keeping the corresponding model predictions fixed. One exploratory classification threshold was chosen from the nested development out-of-fold predictions using the Youden index and then transferred unchanged to the temporal cohort. At that threshold, we calculated accuracy, sensitivity, specificity, positive predictive value (PPV), negative predictive value (NPV), F1 score, and the confusion matrix. The resulting cutoff was regarded as exploratory, not as a general clinical decision threshold.

Clinical usefulness was explored with decision-curve analysis over risk thresholds from 2 to 30% (19). Crossing the threshold was interpreted as eligibility for intensified functional follow-up and/or pelvic-floor rehabilitation support. Net benefit for the model was compared with policies of offering that intensified pathway to all patients or to none. This analysis was used to assess potential decision value only; it was not treated as evidence that model-guided care improves patient outcomes.

### 2.8 Final model specification

To support reproducibility, the locked model was reported as a logistic equation using the development-cohort standardization constants for continuous predictors. The equation and all coefficients are provided in the Results. Because the coefficients were obtained under penalization, they are predictive parameters rather than conventional unpenalized effect estimates; coefficient-specific *p*-values were therefore not used for interpretation.

### 2.9 Software, ethics, and reporting

Analyses were conducted in Python 3.13.5 with scikit-learn 1.8.0, pandas 2.2.3, NumPy 2.3.5, statsmodels 0.14.6, and Matplotlib 3.10.8. Random seeds were fixed to support reproducibility. The Ethics Committee of the Second Affiliated Hospital of Guizhou University of Traditional Chinese Medicine approved the study (approval no. 2026-EC-216). Records were de-identified before analysis, and individual informed consent was waived for this retrospective use of clinical data. Reporting was guided by the TRIPOD+AI statement for prediction models developed with regression or machine-learning methods (20).

## 3 Results

### 3.1 Cohort characteristics

The development cohort consisted of 726 patients with 51 PPI events (7.0%). Of 476 potentially eligible patients identified during the temporal-validation period, 11 were excluded according to the eligibility and data-completeness criteria, leaving 465 patients; 38 of these patients experienced PPI (8.2%). Event prevalence was similar between cohorts, but several aspects of case mix differed modestly, including a greater use of robotic surgery and changes in estimated blood loss and nerve-sparing practice in the later period. Descriptive standardized mean differences are shown in Table 1.

**Table 1:** Characteristics of the development and same-center temporal validation cohorts.

Variable	Development (*n* = 726)	Temporal validation (*n* = 465)	|SMD|
Age, years	65.1 ± 7.8	64.6 ± 7.7	0.066
BMI, kg/m^2^	26.9 ± 3.5	26.7 ± 3.7	0.039
Prostate volume, mL	48.7 ± 16.1	49.5 ± 16.2	0.049
Operative time, min	136.2 ± 32.4	133.8 ± 34.1	0.073
Postoperative hospital stay, days	4.2 ± 1.1	4.1 ± 1.2	0.062
Membranous urethral length, mm	12.8 ± 2.5	12.9 ± 2.6	0.037
Apical urethral injury score	2.1 ± 0.8	2.2 ± 0.9	0.081
Preoperative IPSS	8 (4, 14)	9 (4, 14)	0.015
Preoperative PSA, ng/mL	7.2 (4.8, 11.5)	7.6 (4.5, 12.3)	0.033
Estimated blood loss, mL	150 (100, 250)	130 (80, 220)	0.148
Catheter duration, days	7 (5, 9)	7 (5, 9)	0.018
Smoking history	228 (31.4%)	148 (31.8%)	0.009
Alcohol history	263 (36.2%)	176 (37.8%)	0.034
Hypertension	325 (44.8%)	195 (41.9%)	0.057
Diabetes	115 (15.8%)	79 (17.0%)	0.031
Urination-affecting medication history	94 (12.9%)	55 (11.8%)	0.034
Pelvic lymph-node dissection	290 (39.9%)	202 (43.4%)	0.071
Surgery type	Open 53 (7.3%); laparoscopic 145 (20.0%); robotic 528 (72.7%)	Open 15 (3.2%); laparoscopic 69 (14.8%); robotic 381 (81.9%)	0.221
Neurovascular bundle preservation	Bilateral 497 (68.5%); unilateral 174 (24.0%); none 55 (7.6%)	Bilateral 322 (69.2%); unilateral 122 (26.2%); none 21 (4.5%)	0.129
12-month PPI (ICIQ-SF ≥ 1)	51 (7.0%)	38 (8.2%)	0.043

Values are mean ± SD, median (interquartile range), or *n* (%). For multicategory variables, the largest absolute SMD across indicator categories is shown. SMDs are descriptive and were not interpreted with *p*-value thresholds.

The development cohort contained 51 events for 21 candidate predictor parameters before penalization, corresponding to 2.43 events per candidate predictor parameter. In the primary Riley sensitivity scenario (anticipated Nagelkerke R-squared = 0.15, corresponding to Cox-Snell R-squared approximately 0.060; target shrinkage = 0.90), the minimum required sample sizes were 3,044 participants for the shrinkage criterion, 1,011 for limiting optimism in Nagelkerke R-squared, and 101 for precise estimation of the overall outcome proportion. The governing requirement was therefore approximately 3,044 participants, corresponding to about 214 events at a 7.0% event proportion. Across the broader illustrative Nagelkerke R-squared sensitivity range of 0.10–0.30, the minimum required sample ranged from approximately 1,468 to 4,620 participants (about 103–325 events), and the available cohort remained below the calculated requirement throughout.

**Table 2:** Non-zero coefficients in the final LASSO-penalized logistic model.

Predictor	Scale/coding	Coefficient	exp(β)	Bootstrap retention
Age	per 1 SD (7.84 years)	1.093	2.98	100.0%
Preoperative IPSS	per 1 SD (7.61 points)	0.788	2.20	100.0%
ln(1 + PSA)	per 1 SD (0.664)	0.765	2.15	100.0%
Operative time	per 1 SD (32.35 min)	−0.122	0.89	82.4%
Postoperative hospital stay	per 1 SD (1.14 days)	−0.026	0.97	68.5%
Smoking history	yes vs. no	0.201	1.22	65.7%
Hypertension	yes vs. no	0.173	1.19	65.4%
Unilateral nerve sparing	vs bilateral	1.123	3.07	100.0%*
No nerve sparing	vs bilateral	1.509	4.52	100.0%*

^*^Bootstrap retention for neurovascular bundle preservation was assessed at the original-predictor level, defined by at least one non-zero coefficient for this three-level variable. Penalized coefficients and exp(β) values are prediction parameters and should not be interpreted as conventional inferential odds ratios.

### 3.2 LASSO variable selection and coefficient stability

Repeated cross-validation in the development cohort selected C = 0.427 under the one-standard-error rule. The final full-cohort LASSO fit retained eight original predictors: age, preoperative IPSS, ln(1 + PSA), operative time, postoperative hospital stay, smoking history, hypertension, and neurovascular bundle preservation. The three-level nerve-sparing variable contributed two coefficients, giving nine non-zero predictor coefficients plus the intercept. Age, IPSS, ln(1 + PSA), and nerve-sparing status appeared in all 1,000 bootstrap refits. Retention frequencies were 82.4% for operative time, 68.5% for postoperative hospital stay, 65.7% for smoking history, and 65.4% for hypertension. The first four signals were therefore highly stable, whereas the smaller terms showed greater selection sensitivity (Table 2).

Figure 1 summarizes LASSO variable selection and penalty tuning. Panel A shows the coefficient paths across the regularization sequence, and panel B shows the repeated cross-validated log-loss profile used to select the penalty by the one-standard-error rule.

**Figure 1:**
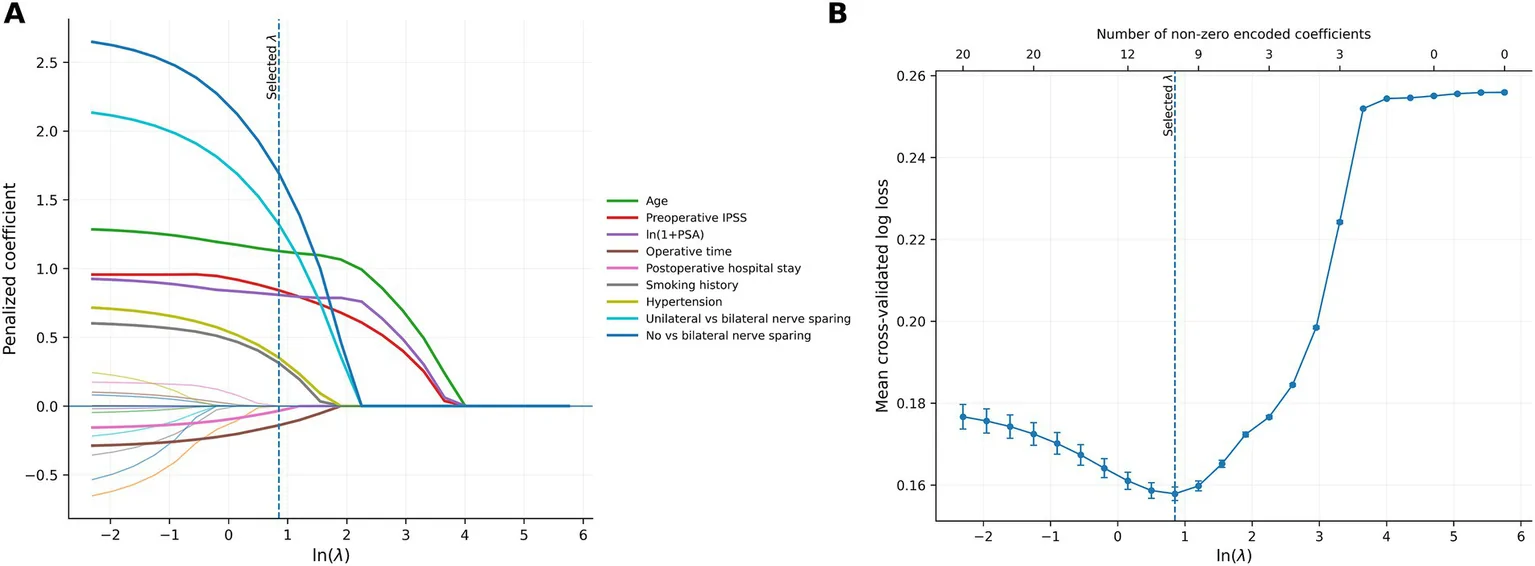
LASSO variable selection and penalty tuning in the development cohort. **(A)** Coefficient paths across the regularization sequence. **(B)** Cross-validated log loss across the candidate penalty grid. The dashed vertical line indicates the penalty selected using the one-standard-error rule; the upper axis in panel B shows the number of non-zero encoded coefficients.

### 3.3 Nested internal comparison of candidate models

Across nested repeated out-of-fold predictions, LASSO produced a numerically higher ROC-AUC and average precision and a lower Brier score than constrained GBM. The nested internal validation performance of the two candidate modeling strategies is summarized in Table 3. LASSO was taken forward because it was numerically favorable on the principal probability-performance measures and provided a sparse, readily interpretable coefficient structure.

**Table 3:** Nested internal out-of-fold performance of the two candidate strategies.

Model	ROC-AUC	Average precision	Brier score	Decision
LASSO-penalized logistic regression	0.913 (0.871–0.946)	0.542 (0.404–0.675)	0.046 (0.035–0.058)	Selected
Constrained GBM	0.896 (0.854–0.936)	0.488 (0.359–0.620)	0.048 (0.037–0.062)	Not selected

Both strategies used the same 19 candidate inputs. LASSO combined shrinkage with embedded variable selection, whereas GBM used the prespecified constrained configuration. Confidence intervals are patient-level bootstrap intervals based on averaged outer out-of-fold predictions.

### 3.4 Temporal performance of the locked LASSO model

In 465 later patients, the locked development model achieved a ROC-AUC of 0.836 (95% CI 0.763–0.900), average precision of 0.409 (0.277–0.564), and a Brier score of 0.062 (0.045–0.081). Average precision remained substantially above the event-prevalence benchmark of 0.082. Mean predicted risk was 5.9%, whereas the observed event rate was 8.2%. Figures 2, 3 present the corresponding ROC and precision-recall curves.

**Figure 2:**
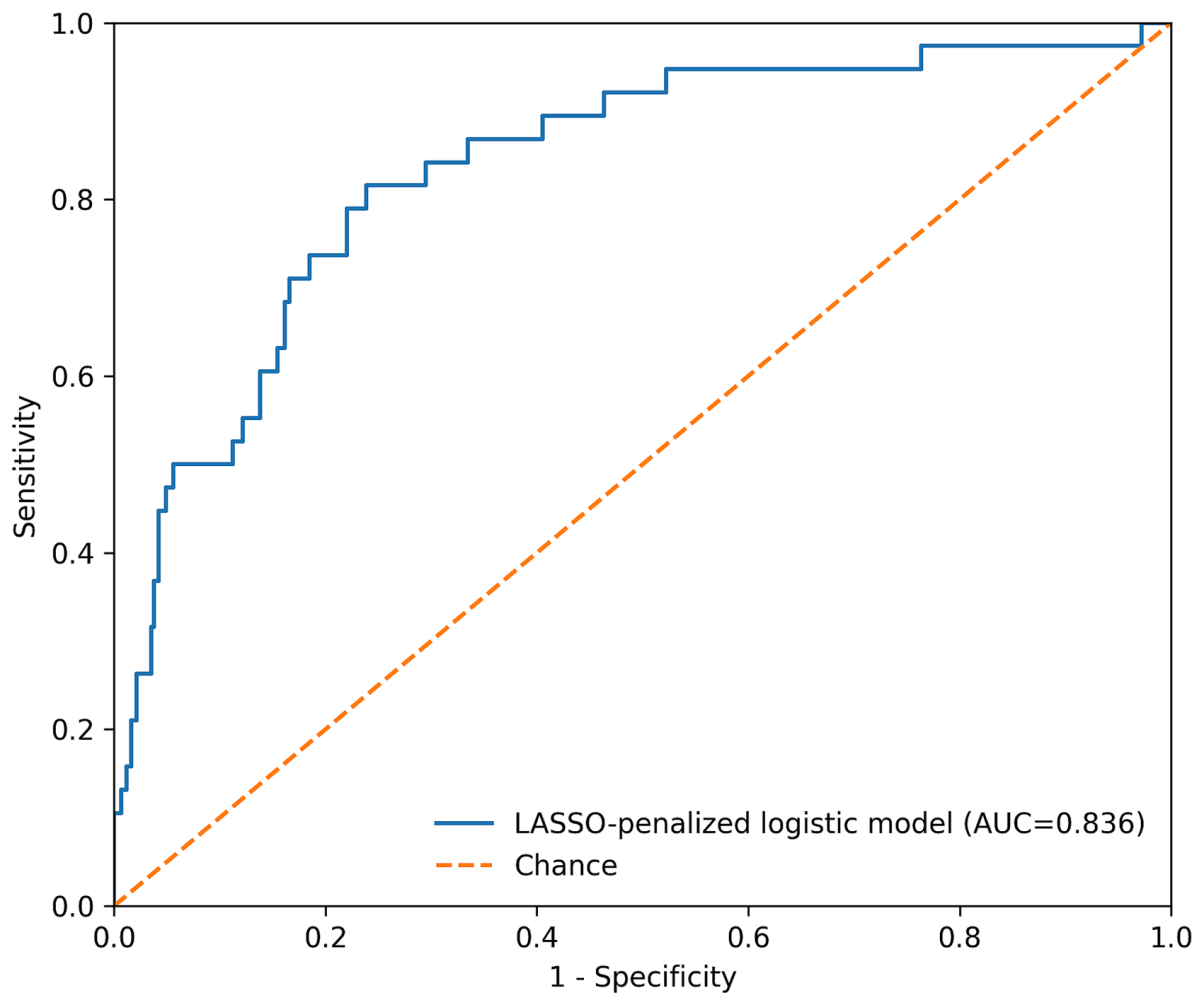
Receiver operating characteristic curve for the locked LASSO model in the same-center temporal cohort. ROC-AUC = 0.836.

**Figure 3:**
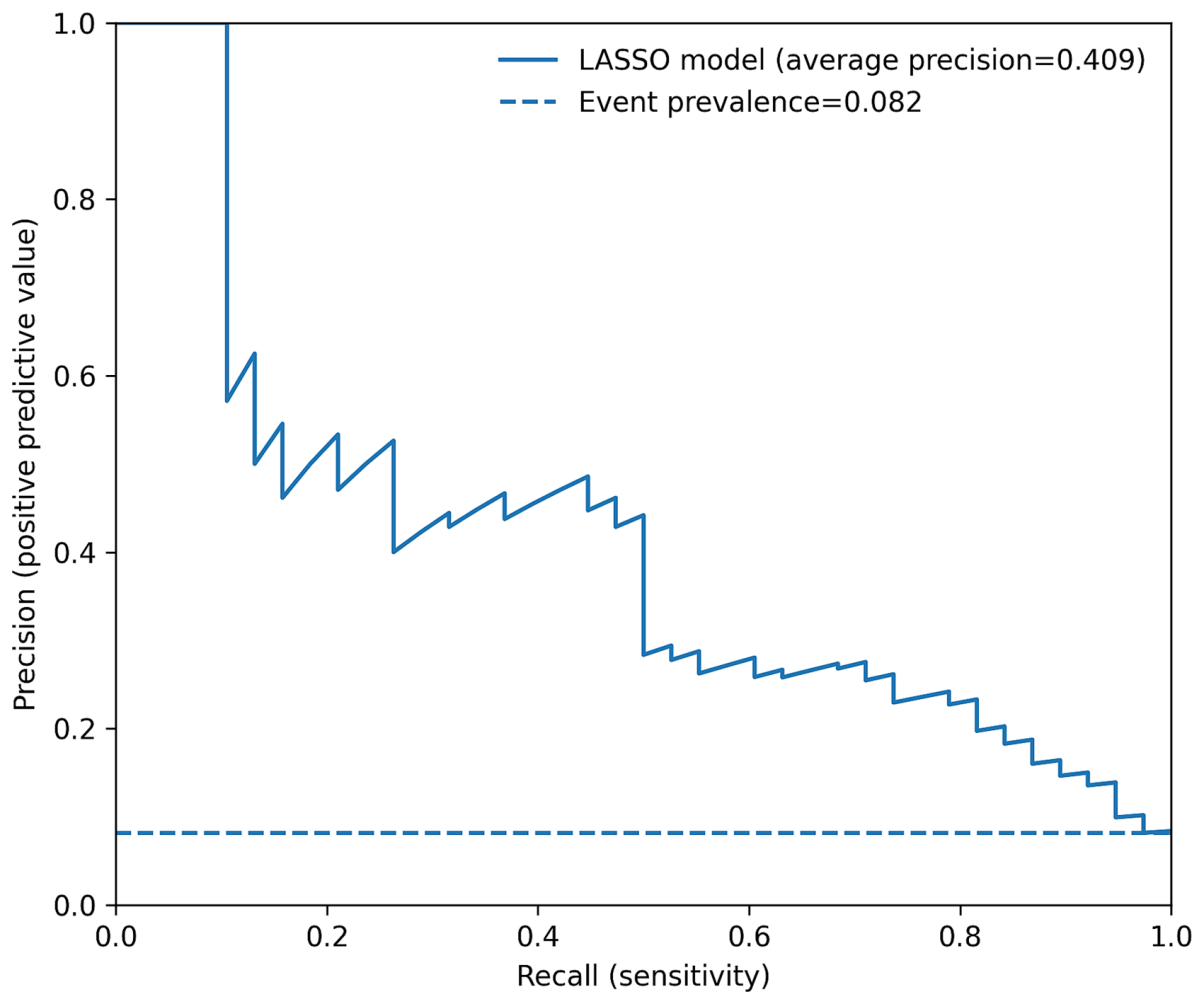
Precision-recall curve for the locked LASSO model in the same-center temporal cohort. Average precision = 0.409; the horizontal reference line marks the 12-month PPI prevalence of 0.082.

CITL was 0.445 (95% CI 0.012–0.808) and the calibration slope was 0.850 (0.621–1.174). Thus, the model continued to separate higher- from lower-risk patients, but it underestimated risk on average in the later cohort. The calibration results do not justify assuming that the original absolute probabilities will transport unchanged to other settings. Figure 4A shows the temporal calibration plot.

**Figure 4:**
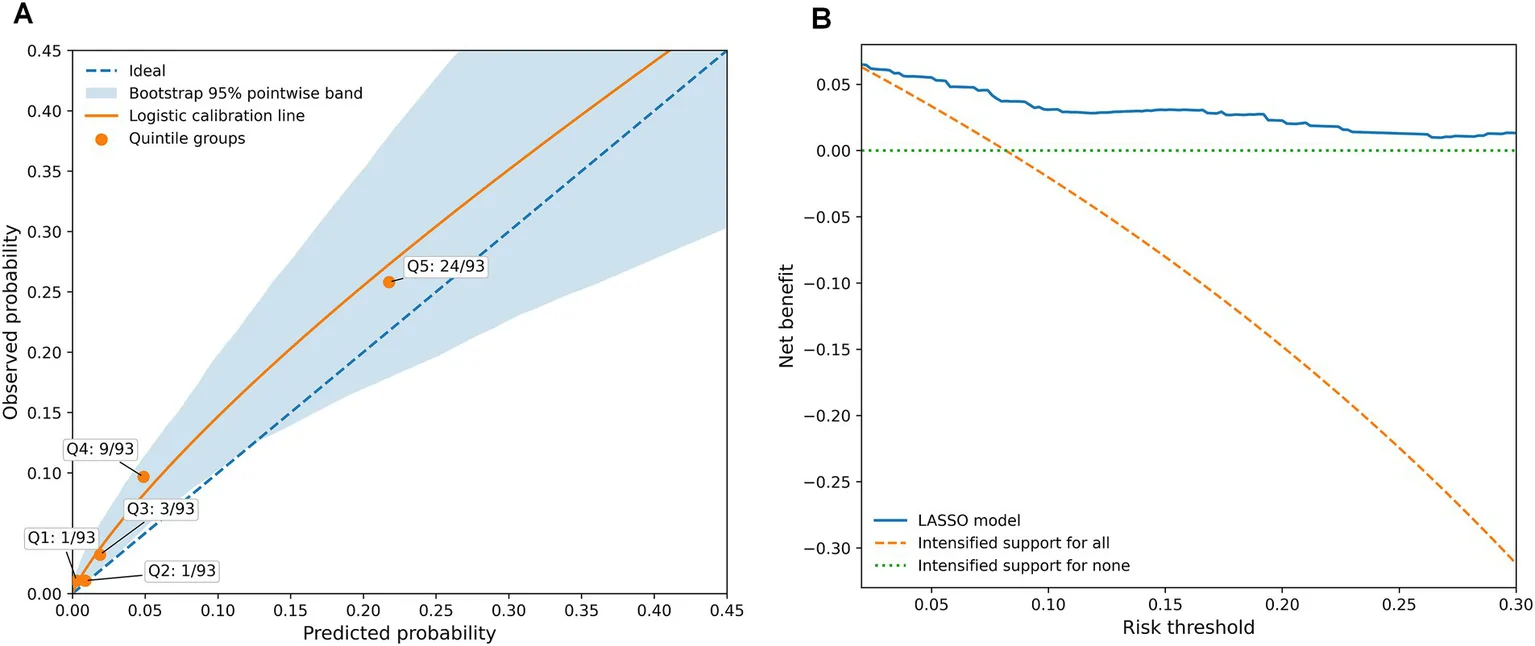
Temporal assessment of calibration and potential clinical utility for the locked LASSO model. **(A)** Calibration: the solid line shows the logistic calibration relation, the shaded area the bootstrap 95% pointwise band, and points the observed versus mean predicted risk by quintile (events/participants); CITL = 0.445 and calibration slope = 0.850. **(B)** Decision-curve analysis: net benefit is displayed for risk thresholds of 2–30% and compared with intensified-support-for-all and intensified-support-for-none strategies. The analysis is retrospective and should not be interpreted as a clinical impact study.

The exploratory Youden cutoff derived from development predictions was 0.128. Applied without re-optimization, it yielded an accuracy of 0.880, sensitivity of 0.500, specificity of 0.913, PPV of 0.339, NPV of 0.954, and F1 score of 0.404 (TP = 19, FP = 37, TN = 390, FN = 19). The marked reduction in sensitivity relative to internal validation shows that the binary cutoff was less stable over time than the underlying continuous risk ranking. The internal and temporal validation performance metrics of the final LASSO model are summarized in Table 4.

**Table 4:** Performance of the final LASSO model in nested internal validation and same-center temporal validation.

Metric	Nested internal OOF	Temporal validation
ROC-AUC	0.913 (0.871–0.946)	0.836 (0.763–0.900)
Average precision	0.542 (0.404–0.675)	0.409 (0.277–0.564)
Brier score	0.046 (0.035–0.058)	0.062 (0.045–0.081)
Calibration-in-the-large	−0.113 (−0.428 to 0.180)	0.445 (0.012–0.808)
Calibration slope	1.365 (1.114–1.690)	0.850 (0.621–1.174)
Exploratory threshold	0.128	0.128 (not re-optimized)
Accuracy	0.873 (0.851–0.898)	0.880 (0.849–0.910)
Sensitivity	0.824 (0.710–0.918)	0.500 (0.333–0.667)
Specificity	0.877 (0.853–0.902)	0.913 (0.886–0.940)
PPV	0.336 (0.261–0.419)	0.339 (0.220–0.472)
NPV	0.985 (0.975–0.993)	0.954 (0.931–0.974)
F1 score	0.477 (0.390–0.565)	0.404 (0.268–0.536)
Confusion matrix	TP = 42, FP = 83, TN = 592, FN = 9	TP = 19, FP = 37, TN = 390, FN = 19

Except for the confusion matrix and fixed threshold, values are point estimates with 95% bootstrap confidence intervals. Internal estimates use averaged outer out-of-fold predictions. The threshold was derived exclusively from development out-of-fold predictions and transferred unchanged to the temporal cohort.

Across thresholds of 2–30%, decision-curve analysis suggested greater net benefit for model-based selection than policies of intensified postoperative follow-up or rehabilitation for all patients or for none. Because these estimates came from retrospective same-center data, they indicate possible decision value rather than established clinical benefit. The decision curve is presented in Figure 4B.

### 3.5 Final model equation and interpretation

The final LASSO-penalized logistic model fitted to all 726 development participants was:

logit[P(PPI)]=−4.2987+1.0927Z(age)+0.7880Z(IPSS)

+0.7648Z[ln(1+PSA)]−0.1218Z(operative time)

$$\begin{array}{l} -0.0260\;Z(\text{postoperative hospital stay})\\ +0.2007\;I(\text{smoking history})+0.1735\;I(\text{hypertension}) \end{array}$$

+1.1230I(unilateral nerve sparing)+1.5087I(nonerve sparing)

Z(age)=(age−65.1405)/7.8405.

Z(IPSS)=(IPSS−10.2397)/7.6124.

Z[ln(1+PSA)]=[ln(1+PSA)−2.1645]/0.6640.

Z(operative time)=(operative time−136.2121)/32.3461.

Z(postoperative hospital stay)=(hospital stay−4.2176)/1.1413.

Bilateral nerve sparing was the reference category. This equation is intended for prediction; coefficient direction and magnitude should not be read as causal effects or conventional hypothesis-test estimates.

## 4 Discussion

### 4.1 Principal findings

This analysis examined whether a parsimonious perioperative model could identify patients at increased risk of persistent PPI when only a limited number of outcome events were available for development. Beyond the algorithmic comparison, the intended public-health relevance lies in whether routinely collected information can support more proportionate survivorship follow-up and rehabilitation planning without adding a substantial assessment burden. LASSO reduced 19 candidate predictors to eight original variables, with age, preoperative IPSS, transformed PSA, and nerve-sparing status showing the strongest selection stability. In repeated nested validation, LASSO had numerically better ROC-AUC, average precision, and Brier score than constrained GBM, but this descriptive comparison does not establish algorithmic superiority. When the locked model was evaluated in patients treated later, discrimination remained clinically informative, but probabilities were too low on average and sensitivity at the development-derived cutoff fell to 0.50. These findings favor use of the model as a graded risk estimate rather than an inflexible binary rule.

The temporal results also emphasize the distinction between ranking and absolute risk estimation for service-oriented use. ROC-AUC declined from about 0.91 in internal validation to 0.84 in the later cohort. Internal and temporal calibration showed different patterns. The internal calibration slope of 1.365 (95% CI 1.114–1.690) indicates that the averaged out-of-fold predictions were less dispersed than ideal, whereas the temporal slope was 0.850 (0.621–1.174), with its confidence interval including 1. These differences reinforce that calibration characteristics were not stable over time and should be interpreted in light of the limited number of outcome events. The temporal cohort had an observed PPI rate of 8.2% compared with a mean predicted risk of 5.9%, despite a lower prevalence of no nerve sparing than in the development cohort. Thus, the average underprediction cannot be explained by nerve-sparing distribution alone. Changes in other measured characteristics, unmeasured aspects of surgical or postoperative care, outcome ascertainment, or predictor–outcome relationships may have contributed to the temporal shift in baseline risk. For postoperative counseling, rehabilitation prioritization, and planning of follow-up intensity, this matters because decisions based on predicted probabilities require acceptable calibration, not discrimination alone (12, 13, 15). The observed temporal shift therefore argues against transferring absolute risks or a single binary threshold to another service setting without local assessment.

### 4.2 Model complexity, LASSO selection, and comparison with gradient boosting

The comparison with GBM was designed to address a practical modeling question rather than to identify a universally superior algorithm. Evidence from clinical prediction research shows that greater computational flexibility does not automatically translate into better out-of-sample performance (9). In the present development cohort, only 51 events were available for 19 candidate predictors representing 21 candidate predictor parameters before penalization, making uncontrolled model complexity particularly undesirable (11). LASSO offered shrinkage and a compact coefficient structure, whereas GBM was intentionally restricted. The absence of a clear predictive gain from GBM therefore supports parsimony in this dataset; it should not be generalized as evidence against gradient boosting in larger or richer datasets.

The retained variables also require a predictive rather than etiologic interpretation. Age, IPSS, transformed PSA, and nerve-sparing status were selected in essentially every bootstrap refit, whereas operative time, postoperative stay, smoking, and hypertension appeared less consistently and carried smaller penalized coefficients. Correlation among predictors and competition among weak signals can make LASSO selections unstable when events are sparse (10). Accordingly, the smaller coefficients are best understood as components of the fitted prediction function, not as proof of independent biological effects.

Membranous urethral length and apical urethral injury score were included among the original candidate predictors but were not retained in the final penalized fit. Their non-selection should not be interpreted as evidence that these anatomical features are clinically unimportant. In LASSO modeling, correlated predictors can compete to represent shared predictive information, and weaker signals may be excluded when outcome events are limited or when stronger penalization is applied. Measurement variability and the distribution of these features in the present cohort may also contribute. We therefore regard their omission as dataset- and penalty-dependent rather than as evidence against their previously reported clinical relevance.

### 4.3 Clinical interpretation of retained predictors

Among the more stable predictors, older age and greater preoperative urinary symptom burden were associated with higher estimated PPI risk. The age signal is directionally consistent with prospective and longer-term studies reporting slower continence recovery among older patients (21, 22). Possible explanations include differences in sphincter reserve, pelvic-floor function, neuromuscular control, and recovery capacity, but the present study was not designed to identify causal pathways.

Preoperative IPSS provides a practical summary of baseline lower urinary tract symptoms, and greater symptom burden has been associated with poorer postoperative functional recovery and health-related quality of life (23). Transformed PSA also contributed to prediction, although it may be acting as a marker of disease burden, anatomy, or procedural complexity rather than as a direct determinant of continence. The largest categorical coefficients were observed for incomplete neurovascular bundle preservation, consistent in direction with previous work relating nerve-sparing techniques to continence recovery (24–26). Because nerve-sparing status is finalized perioperatively, the model should be viewed as an updated perioperative risk estimate rather than a purely preoperative calculator.

Operative time and postoperative hospital stay had small negative penalized coefficients. We verified the source-variable units and coding direction: operative time was recorded in minutes and postoperative stay in days, with higher values representing longer duration, and neither variable was reverse-coded. Examination of the LASSO coefficient path showed that both variables entered with negative coefficients across the relevant penalty range. These terms are conditional components of a penalized multivariable prediction function and should not be interpreted as causal effects. Their direction may reflect correlation and competition among predictors, shrinkage under penalization, and instability associated with the limited number of outcome events. Their lower bootstrap retention frequencies further support caution in assigning these smaller coefficients stable clinical meaning. Smoking history and hypertension also made smaller contributions and were retained less consistently in bootstrap refits. Replication in independent data will be needed to determine whether these terms remain useful predictive adjustments outside the development setting.

### 4.4 Temporal transportability, calibration, and threshold behavior

Temporal testing is valuable because it exposes a completed model to changes that arise after development, but it cannot establish broad transportability across institutions (15). The later cohort differed modestly in surgical approach and perioperative characteristics, and both discrimination and calibration changed. We deliberately did not recalibrate the model before evaluation, because doing so would have concealed the extent of temporal miscalibration. If future geographic validation shows preserved ranking with a systematic calibration offset, transparent local recalibration may be reasonable before implementation (13, 27).

Threshold behavior was less stable than continuous risk estimation. Although specificity remained high, sensitivity at the fixed 0.128 cutoff fell from approximately 0.82 internally to 0.50 in the temporal cohort. An empirically optimized cutoff can be sensitive to changes in case mix and outcome prevalence, even when the model continues to rank patients reasonably well (12, 15). The present threshold should therefore be considered descriptive. Any intervention threshold used in practice would need to reflect the consequences of missed high-risk patients, the burden of additional follow-up or rehabilitation, and available service capacity rather than the Youden criterion alone (19).

Decision-curve analysis suggested potential net benefit over the examined range (19). The 2–30% range was examined to characterize decision behavior over a broad range of low-to-moderate predicted risks and should not be interpreted as implying that every threshold in this interval is clinically actionable. No intervention threshold was prospectively prespecified or validated in this study. For the intended use of intensified functional follow-up and/or pelvic-floor rehabilitation, thresholds toward the lower portion of the examined range are likely to be more relevant than the upper end because the proposed intervention carries substantially less burden than an invasive treatment decision. The exact actionable threshold should nevertheless be defined prospectively according to the relative consequences of missed persistent incontinence, additional follow-up or rehabilitation, patient preferences, and local service capacity. Positive net benefit indicates potential decision value but does not establish that model-guided care improves patient outcomes. A prospective impact study would be required to assess whether risk-informed counseling, rehabilitation intensity, or follow-up schedules improve continence, quality of life, adherence, resource utilization, or cost-effectiveness (27).

### 4.5 Public-health implications for survivorship care and service planning

Persistent PPI is relevant beyond the immediate surgical episode because it can impair day-to-day function and sustain demand for continence products, nursing input, rehabilitation, and outpatient services (2, 28). From a public-health survivorship and health-service perspective, a risk tool based on routinely collected perioperative data could help identify patients who may need earlier or more structured functional support without adding a substantial assessment burden (28). Potential uses include risk-informed communication, prioritization of pelvic-floor rehabilitation, and planning the intensity of functional follow-up. These are service-planning applications rather than evidence that the model itself improves outcomes, and the tool should not determine surgical eligibility, mandate nerve-sparing decisions, substitute for clinical judgment, or operate as a stand-alone gatekeeper to care.

Implementation would also require attention to transportability. The later cohort showed average underprediction, and the fixed development threshold lost sensitivity despite preserved discrimination. Accordingly, absolute probabilities should be checked and, where appropriate, recalibrated locally before they are used to prioritize care (13, 27). Service thresholds should also reflect the consequences of missed high-risk patients, the burden and availability of additional follow-up or rehabilitation, and local capacity rather than the Youden criterion alone (19). Independent geographic validation and prospective impact evaluation are needed to determine whether risk-informed pathways improve functional outcomes, patient experience, adherence, resource use, or cost-effectiveness before the model can support routine public-health or health-service practice.

### 4.6 Strengths and limitations

Several design features strengthen the analysis. All available development patients were retained rather than sacrificing events to a single random holdout. Preprocessing, LASSO tuning, and variable selection were nested within resampling, and the final comparison included a constrained nonlinear alternative evaluated on the same starting predictors. Performance assessment extended beyond ROC-AUC to precision-recall performance, Brier score, calibration, threshold-dependent measures, and decision-curve analysis. Finally, the completed LASSO model was carried forward to a non-overlapping later cohort without coefficient updating or recalibration in the revised analysis pipeline.

The study also has important limitations. First, both cohorts were retrospective and came from one clinical setting, so performance across hospitals, regions, and surgical teams remains unknown. Second, only 51 events were available in development and 38 in temporal evaluation. The number of outcome events was limited relative to the candidate predictor space, which comprised 21 candidate predictor parameters before penalization. Although LASSO shrinkage reduces effective model complexity, it cannot eliminate uncertainty in variable selection or coefficient estimation when outcome events are sparse. This concern is reflected in the bootstrap analysis: the strongest predictors were retained consistently, whereas several smaller terms showed only moderate retention frequencies. These less stable coefficients should therefore be regarded as provisional predictive adjustments rather than robust independent effects. Penalization and nested resampling can reduce overfitting but cannot compensate for limited outcome information. Consistent with this concern, the *post hoc* Riley assessment indicated a minimum of approximately 3,044 participants and 214 events in the primary conservative sensitivity scenario, versus the 726 participants and 51 events available; the exact requirement is assumption-dependent. The final predictor set and the smaller penalized coefficients should therefore be regarded as provisional pending evaluation in larger independent datasets (see Section 4.2).

The small number of events also affected internal validation. With five-fold outer cross-validation, each outer validation fold contained approximately 10 PPI events on average. Repetition reduces dependence on a particular random partition but does not add independent outcome information. Internal discrimination, calibration, and threshold-dependent estimates therefore remain subject to appreciable sampling variability. This uncertainty should be considered alongside temporal changes in case mix, baseline risk, and clinical practice when interpreting the lower performance observed in the later cohort.

Third, analysis required complete 12-month outcome ascertainment. Because a full comparison between patients with and without completed follow-up was unavailable, selection related to attrition cannot be excluded. Fourth, the temporal cohort had been inspected during an earlier modeling stage, limited to baseline descriptive characteristics, outcome prevalence, basic data-quality indicators, and exploratory performance outputs from a preliminary model. Although these observations were not used to estimate, tune, update, or recalibrate the final revised LASSO model, indirect influence from prior awareness cannot be completely excluded. Accordingly, this evaluation should not be interpreted as a fully untouched confirmatory validation. Independent validation in a new center or in a genuinely future cohort remains necessary.

Fifth, PPI was defined as ICIQ-SF ≥ 1 at 12 months after catheter removal. The ICIQ-SF is validated (16), but this study-specific threshold was chosen to capture any residual self-reported symptom and is not an established universal diagnostic cutoff; continence definitions after prostatectomy vary across the literature (3). A parallel pad-based outcome was not available for sensitivity analysis. Sixth, candidate predictors were limited to variables present in the source database, so additional anatomic, technical, behavioral, or rehabilitation factors may provide useful information. Finally, decision-curve analysis was retrospective and cannot establish that model-guided care improves outcomes (19, 27).

## 5 Conclusion

Using routinely available perioperative information in a low-event development cohort, LASSO produced an eight-predictor model that was more parsimonious and numerically better on internal probability-performance measures than a constrained GBM. The locked model retained useful discrimination when evaluated in patients treated later, but average risk was underestimated and the fixed development threshold had limited sensitivity. These findings support continued evaluation of the model as a continuous aid to risk-informed survivorship follow-up and rehabilitation planning rather than immediate use as a stand-alone binary decision rule. Before use to prioritize supportive-care or rehabilitation resources in routine practice, geographic external validation, setting-specific calibration assessment, and prospective impact evaluation are required.

## Data Availability

The datasets presented in this article are not readily available because the dataset contains protected clinical information and is subject to institutional ethics and data-governance requirements. Access may be considered upon reasonable request to the corresponding author, subject to approval by the responsible institution and applicable data-use requirements. Requests to access the datasets should be directed to 1095649731@qq.com.

## References

[1] Mottet N van den Bergh RCN Briers E Van den Broeck T Cumberbatch MG De Santis M . EAU-EANM-ESTRO-ESUR-SIOG guidelines on prostate cancer—2020 update. Part 1: screening, diagnosis, and local treatment with curative intent. Eur Urol. (2021) 79:243–62. doi: 10.1016/j.eururo.2020.09.042, 33172724

[2] Resnick MJ Koyama T Fan KH Albertsen PC Goodman M Hamilton AS . Long-term functional outcomes after treatment for localized prostate cancer. N Engl J Med. (2013) 368:436–45. doi: 10.1056/NEJMoa1209978, 23363497 PMC3742365

[3] Ficarra V Novara G Rosen RC Artibani W Carroll PR Costello A . Systematic review and meta-analysis of studies reporting urinary continence recovery after robot-assisted radical prostatectomy. Eur Urol. (2012) 62:405–17. doi: 10.1016/j.eururo.2012.05.045, 22749852

[4] Koss Modig K Arnsrud Godtman R Carlsson S Stattin P Styrke J Månsson M . Patient- and procedure-specific risk factors for urinary incontinence after robot-assisted radical prostatectomy: a nationwide, population-based study. Eur Urol Oncol. (2025) 8:932–40. doi: 10.1016/j.euo.2025.03.015, 40307091

[5] Huang H Cai K. Prognostic factors for urinary incontinence after robot-assisted radical prostatectomy: a systematic review and meta-analysis. J Robot Surg. (2025) 19:628. doi: 10.1007/s11701-025-02803-6, 40987992

[6] Amparore D De Cillis S Alladio E Sica M Piramide F Verri P . Development of machine learning algorithm to predict the risk of incontinence after robot-assisted radical prostatectomy. J Endourol. (2024) 38:871–8. doi: 10.1089/end.2024.0057, 38512711

[7] Park S Byun J. A study of predictive models for early outcomes of post-prostatectomy incontinence: machine learning approach vs. logistic regression analysis approach. Appl Sci (Basel). (2021) 11:6225. doi: 10.3390/app11136225

[8] Hung AJ Chen J Ghodoussipour S Oh PJ Liu Z Nguyen J . A deep-learning model using automated performance metrics and clinical features to predict urinary continence recovery after robot-assisted radical prostatectomy. BJU Int. (2019) 124:487–95. doi: 10.1111/bju.14735, 30811828 PMC6706286

[9] Christodoulou E Ma J Collins GS Steyerberg EW Verbakel JY Van Calster B. A systematic review shows no performance benefit of machine learning over logistic regression for clinical prediction models. J Clin Epidemiol. (2019) 110:12–22. doi: 10.1016/j.jclinepi.2019.02.004, 30763612

[10] Tibshirani R. Regression shrinkage and selection via the lasso. J R Stat Soc Ser B Stat Methodol. (1996) 58:267–88. doi: 10.1111/j.2517-6161.1996.tb02080.x

[11] Riley RD Snell KIE Ensor J Burke DL Harrell FE Jr Moons KGM . Minimum sample size for developing a multivariable prediction model: PART II—binary and time-to-event outcomes. Stat Med. (2019) 38:1276–96. doi: 10.1002/sim.7992 PMC6519266 30357870

[12] Steyerberg EW Vickers AJ Cook NR Gerds T Gonen M Obuchowski N . Assessing the performance of prediction models: a framework for traditional and novel measures. Epidemiology. (2010) 21:128–38. doi: 10.1097/EDE.0b013e3181c30fb2, 20010215 PMC3575184

[13] Van Calster B McLernon DJ van Smeden M Wynants L Steyerberg EW Topic Group ‘Evaluating diagnostic tests and prediction models’ of the STRATOS initiative. Calibration: the Achilles heel of predictive analytics. BMC Med. (2019) 17:230. doi: 10.1186/s12916-019-1466-7 PMC6912996 31842878

[14] Saito T Rehmsmeier M. The precision-recall plot is more informative than the ROC plot when evaluating binary classifiers on imbalanced datasets. PLoS One. (2015) 10:e0118432. doi: 10.1371/journal.pone.0118432, 25738806 PMC4349800

[15] Steyerberg EW Harrell FE Jr. Prediction models need appropriate internal, internal-external, and external validation. J Clin Epidemiol. (2016) 69:245–7. doi: 10.1016/j.jclinepi.2015.04.005, 25981519 PMC5578404

[16] Avery K Donovan J Peters TJ Shaw C Gotoh M Abrams P. ICIQ: a brief and robust measure for evaluating the symptoms and impact of urinary incontinence. Neurourol Urodyn. (2004) 23:322–30. doi: 10.1002/nau.20041, 15227649

[17] Friedman JH. Greedy function approximation: a gradient boosting machine. Ann Stat. (2001) 29:1189–232. doi: 10.1214/aos/1013203451

[18] Varma S Simon R. Bias in error estimation when using cross-validation for model selection. BMC Bioinformatics. (2006) 7:91. doi: 10.1186/1471-2105-7-91, 16504092 PMC1397873

[19] Vickers AJ Elkin EB. Decision curve analysis: a novel method for evaluating prediction models. Med Decis Mak. (2006) 26:565–74. doi: 10.1177/0272989X06295361, 17099194 PMC2577036

[20] Collins GS Moons KGM Dhiman P Riley RD Beam AL Van Calster B . TRIPOD+AI statement: updated guidance for reporting clinical prediction models that use regression or machine learning methods. BMJ. (2024) 385:e078378. doi: 10.1136/bmj-2023-078378, 38626948 PMC11019967

[21] Gondoputro W Thompson J Evans M Bolton D Frydenberg M Murphy DG . How does age affect urinary continence following robot-assisted radical prostatectomy? A prospective multi-institutional study using independently collected, validated questionnaires. J Urol. (2022) 207:1048–56. doi: 10.1097/JU.0000000000002391, 34978202

[22] Cano Garcia C Wenzel M Humke C Wittler C Dislich J Incesu RB . Impact of age on long-term urinary continence after robotic-assisted radical prostatectomy. Medicina (Kaunas). (2023) 59:1153. doi: 10.3390/medicina59061153, 37374357 PMC10305537

[23] Westhofen T Feyerabend E Buchner A Schlenker B Becker A Eismann L . Impact of preoperative LUTS on health-related quality of life following radical prostatectomy: a propensity score matched longitudinal study. Urology. (2024) 192:52–8. doi: 10.1016/j.urology.2024.04.020, 38679296

[24] Reeves F Preece P Kapoor J Everaerts W Murphy DG Corcoran NM . Preservation of the neurovascular bundles is associated with improved time to continence after radical prostatectomy but not long-term continence rates: results of a systematic review and meta-analysis. Eur Urol. (2015) 68:692–704. doi: 10.1016/j.eururo.2014.10.020, 25454614

[25] Kim M Park M Pak S Choi SK Shim M Song C . Integrity of the urethral sphincter complex, nerve-sparing, and long-term continence status after robotic-assisted radical prostatectomy. Eur Urol Focus. (2019) 5:823–30. doi: 10.1016/j.euf.2018.04.021, 29759661

[26] Katsimperis S Juliebø-Jones P Ta A Tandogdu Z Al-Bermani O Bellos T . Surgical techniques to preserve continence after robot-assisted radical prostatectomy. Front Surg. (2023) 10:1289765. doi: 10.3389/fsurg.2023.1289765, 38026481 PMC10655003

[27] Moons KGM Kengne AP Grobbee DE Royston P Vergouwe Y Altman DG . Risk prediction models: II. External validation, model updating, and impact assessment. Heart. (2012) 98:691–8. doi: 10.1136/heartjnl-2011-301247, 22397946

[28] Anderson CA Omar MI Campbell SE Hunter KF Cody JD Glazener CMA. Conservative management for postprostatectomy urinary incontinence. Cochrane Database Syst Rev. (2015) 2015:CD001843. doi: 10.1002/14651858.CD001843.pub5, 25602133 PMC7025637

